# Spatially Resolved Transcriptomics Technology Facilitates Cancer Research

**DOI:** 10.1002/advs.202302558

**Published:** 2023-08-26

**Authors:** Qian Wang, Yuan Zhi, Moxin Zi, Yongzhen Mo, Yumin Wang, Qianjin Liao, Shanshan Zhang, Zhaojian Gong, Fuyan Wang, Zhaoyang Zeng, Can Guo, Wei Xiong

**Affiliations:** ^1^ NHC Key Laboratory of Carcinogenesis and Hunan Key Laboratory of Cancer Metabolism Hunan Cancer Hospital and the Affiliated Cancer Hospital of Xiangya School of Medicine Central South University Changsha Hunan 410008 P. R. China; ^2^ Key Laboratory of Carcinogenesis and Cancer Invasion of the Chinese Ministry of Education Cancer Research Institute Central South University Changsha Hunan 410008 P. R. China; ^3^ Department of Oral and Maxillofacial Surgery The Second Xiangya Hospital of Central South University Changsha Hunan 410012 P. R. China; ^4^ Department of Otolaryngology Head and Neck Surgery Xiangya Hospital Central South University Changsha Hunan 410008 P. R. China

**Keywords:** spatially resolved transcriptomics (SRT), tumor heterogeneity, cancer‐associated fibroblast (CAF), tumor immune microenvironment, tertiary lymphoid structure (TLS)

## Abstract

Single cell RNA sequencing (scRNA‐seq) provides a great convenience for studying tumor occurrence and development for its ability to study gene expression at the individual cell level. However, patient‐derived tumor tissues are composed of multiple types of cells including tumor cells and adjacent non‐malignant cells such as stromal cells and immune cells. The spatial locations of various cells in situ tissues plays a pivotal role in the occurrence and development of tumors, which cannot be elucidated by scRNA‐seq alone. Spatially resolved transcriptomics (SRT) technology emerges timely to explore the unrecognized relationship between the spatial background of a particular cell and its functions, and is increasingly used in cancer research. This review provides a systematic overview of the SRT technologies that are developed, in particular the more widely used cutting‐edge SRT technologies based on next‐generation sequencing (NGS). In addition, the main achievements by SRT technologies in precisely unveiling the underappreciated spatial locations on gene expression and cell function with unprecedented high‐resolution in cancer research are emphasized, with the aim of developing more effective clinical therapeutics oriented to a deeper understanding of the interaction between tumor cells and surrounding non‐malignant cells.

## Graphical abstract

Spatially resolved transcriptomics (SRT) technology is increasingly used in cancer research because of its unique advantage of obtaining spatial information. This review presents the principles of SRT technologies, their uniqueness in understanding the spatial effects of tumor, stromal, immune cells, and their interactions, thus providing new theoretical support in identifying novel biomarkers and therapeutic targets for cancer prevention and treatment.

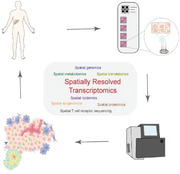

## 1 Introduction

Cancer poses a great burden to society because of its high morbidity and mortality.^[^ ^1^ ^]^ It is imperative to gain insight into the inner workings of tumors in order to propose effective cancer prevention measures and cancer cure strategies. Traditional therapies targeting cancer cells and emerging immunotherapy are not all effective because of individual patient differences and the complex cell niche in which cancer cells reside.^[^ ^2^ ^]^

Solid tumor tissue is a complex ecosystem mainly composed of tumor cells, stromal cells, and various infiltrating immune cells, which control tumor growth and response to therapy relying on their interactions with other cells and the surrounding environment.^[^ ^3^ ^]^ Single‐cell technologies represented by single‐cell RNA sequencing (scRNA‐seq) can analyze the composition and characteristics of cell subpopulations in tumors with an unprecedented high resolution,^[^ ^4^, ^5^ ^]^ but no explanation can be provided for how these different types and states of cells are distributed in space, how they function, and what roles they play.

To better interpret the relationship between tumor cells and their surrounding microenvironment, researchers have tried to understand the occurrence and development of tumor in a spatial dimension. Based on imaging or next‐generation sequencing (NGS) technology,^[^ ^6^ ^]^ spatially resolved transcriptomics (SRT) technology can provide spatial information along with information about the transcriptome of tumor cells and their surrounding microenvironment. This can help us to resolve the spatial structure of tumor microenvironment (TME) in more dimensions, which significantly improves our understanding of cell‐cell interactions and spatial effects in the TME. The SRT technology based on NGS is now widely used in life sciences, especially in cancer research.^[^ ^7^, ^8^, ^9^, ^10^ ^]^ It can characterize the specific cell phenotypes of tumor cells, stromal cells, and infiltrating immune cells in space with their functional counterparts, and identify tertiary lymphoid structure (TLS) existing in the TME, thus advancing immunotherapy and new tumor therapies targeting the microenvironment.

## 2 The SRT Technology

Spatially resolved transcriptomics is a general term used to describe methods for studying gene expression in tissues or cells on a spatial scale. Depending on their detection strategies, SRT technologies can be broadly classified into two categories, imaging‐based methods and NGS‐based methods. **Table** **1** lists SRT technologies of both categories, along with a comparison of their resolution, readout methods, whether they are targeted, and the applicable tissues.

**Table 1:** List of SRT technologies.

Classification	SRT Technology	Resolution	Read‐out	Approach	Tissue Preparation	References
Imaging‐based SRT technology	SeqFISH	Subcellular	Cyclic imaging	Targeted	FF	[12]
	MERFISH	Subcellular	Cyclic imaging	Targeted	FF	[13]
	osmFISH	Subcellular	Cyclic imaging	Targeted	FF	[14]
	seqFISH+	Subcellular	Cyclic imaging	Targeted	FF	[15]
	EEL FISH	Cellular	Cyclic imaging	Targeted	FF	[16]
	BaristaSeq	Subcellular	Sequencing by ligation	Targeted	Cell cultures	[24]
	FISSEQ	Subcellular	Sequencing by ligation	Untargeted	FF/FFPE	[26]
	STARmap	Subcellular	Sequencing by ligation	Targeted	FF	[27]
	HybISS	Cellular	Sequencing by ligation	Targeted	FF	[28]
	ExSeq	Cellular	Sequencing by ligation	Targeted/Untargeted	FF	[29]
NGS‐based SRT technology	10x Visium	Multicellular	NGS	Untargeted	FF/FFPE	[35]
	NanoString GeoMx	Multicellular	NGS	Targeted	FF/FFPE	[38]
	Tomo‐seq	Multicellular	NGS	Untargeted	FF	[44]
	LCM‐seq	Cellular	NGS	Untargeted	FF	[45]
	Geo‐seq	Multicellular	NGS	Untargeted	FF	[46]
	HDST	Subcellular	NGS	Untargeted	FF	[47]
	Slide‐seq	Subcellular to cellular	NGS	Untargeted	FF	[49]
	Slide‐seqV2	Subcellular to cellular	NGS	Untargeted	FF	[54]
	Sci‐Space	Cellular	NGS	Untargeted	FF	[55]
	Seq‐Scope	Subcellular	NGS	Untargeted	FF	[56]
	DBiT‐seq	Cellular	NGS	Untargeted	FF/FFPE	[33]
	Stereo‐seq	Subcellular	NGS	Untargeted	FF	[58]
	Light‐Seq	Cellular	NGS	Untargeted	FF	[59]

SRT: Spatially resolved transcriptomics; NGS: Next‐generation sequencing; FF: Fresh‐frozen; FFPE: Formalin fixation and paraffin embedding.

### 2.1 Imaging‐Based SRT Technology

The exploration of SRT can be traced back to the 1970s,^[^ ^11^ ^]^ which was achieved by in situ hybridization (ISH). Exogenous nucleic acid probes with special labels are used to specifically bind to target RNA molecules to form nucleic acid hybridization molecules, and the position of the nucleic acid to be tested on tissues, cells, or chromosomes is revealed by the corresponding detection means. Based on single‐molecule fluorescence in situ hybridization (smFISH), scientists have developed a series of SRT technologies with new features, including SeqFISH,^[^ ^12^ ^]^ MERFISH,^[^ ^13^ ^]^ osmFISH,^[^ ^14^ ^]^ seqFISH+,^[^ ^15^ ^]^ and EEL FISH,^[^ ^16^ ^]^ which have been improved in different aspects such as imaging area, cost, detection elapsed time, resolution, throughput, and signal‐to‐noise ratio. Even though almost the entire transcriptome can be analyzed,^[^ ^17^ ^]^ it is obvious that ISH techniques must be clear about the target transcript in advance when performing transcript spatialization and are therefore often limited by the target probe library.^[^ ^18^ ^]^ It is also difficult to balance between their target throughput, field of view size, and cost,^[^ ^19^ ^]^ and they are suitable for fixed cells rather than live cells.^[^ ^20^ ^]^

In situ sequencing (ISS)^[^ ^21^ ^]^ is another imaging‐based SRT method. RNA from tissues is reverse transcribed into cDNA, hybridized with a target‐specific padlock probe to form a circular template, and amplified by rolling‐circle amplification (RCA) to generate micrometer‐sized RCA products (RCPs). The target RNA is then identified by decoding the RCP nucleotide sequence using sequencing‐by‐ligation,^[^ ^22^ ^]^ which in turn generates a highly multilayered in situ expression profiles at micron spatial resolution. ISS allows the detection of non‐coding RNAs and microRNAs, but the cumbersome process, high cost, and relatively few targets that can be addressed are its drawbacks.^[^ ^23^ ^]^ BaristaSeq^[^ ^24^ ^]^ is optimized for padlock probe‐based in situ targeted technology and compatible with Illumina sequencing chemistry, which can greatly improve signal‐to‐noise ratio and sequencing accuracy. A non‐targeted technology,^[^ ^25^ ^]^ FISSEQ^[^ ^26^ ^]^ can distinguish up to 8,000 different RNAs, but it produces far fewer reads than RNA‐seq. In contrast, STARmap^[^ ^27^ ^]^ ensures specific amplification of only the target signal and can analyze complete tissue samples in 3D. There is also no limit to the number of RNA species that can be quantitatively accessed, but the complexity of the probe design limits its widespread use. HybISS^[^ ^28^ ^]^ has improved and augmented the use of probes, thus achieving a comprehensive improvement of throughput efficiency, resolution, flexibility, multiplex, and signal‐to‐noise ratio. ExSeq^[^ ^29^ ^]^ combines expansion microscopy and ISS in both targeted and untargeted versions, enabling highly multiplexed imaging of subcellular and even nanoscale transcripts with spatial precision. However, the ISS‐based SRT technology is technically challenging due to current hardware limitations and difficulties in image analysis, and the method has limited detection efficiency, which may lead to the neglect of low‐expressed transcripts.^[^ ^30^ ^]^

In general, both ISH and ISS technologies are used to image RNA molecules in situ for spatial visualization. In the last decades, in situ methods have played an important role in integrating biological functions and spatial information in cancer studies.^[^ ^31^ ^]^ These technologies, combined with image analysis and mathematical modeling, have provided unprecedented understanding of gene expression mechanisms.^[^ ^32^ ^]^ However, it is undeniable that they all have many limitations, in terms of the target range, field of view, and sensitivity of detection.^[^ ^19^ ^]^ In addition, the lengthy repetitive imaging workflows, the high sensitivity required for fluorescent imaging systems, and complex image processing limit their application to the entire transcriptome level.^[^ ^33^ ^]^

### 2.2 NGS‐Based SRT Technology

Fortunately, NGS technology, which is mainly characterized by high throughput and resolution, offers a new approach. The NGS‐based SRT approach relies on adding spatial barcodes^[^ ^6^ ^]^ prior to library construction, collecting these cDNAs with spatial barcodes for sequencing. The barcode of each sequence is used to map the spatial location,^[^ ^34^ ^]^ while the rest is mapped to the genome to identify the transcript source, collectively generating a gene expression matrix. This novel approach avoids the typical limitations of direct visualization and enables unbiased analysis of the complete transcriptome. Several NGS‐based SRT technologies such as 10x Visium and NanoString GeoMx have been developed one after another, applied in developer labs and even commercialized already.

#### 2.2.1 10x Visium

**Figure 1:**
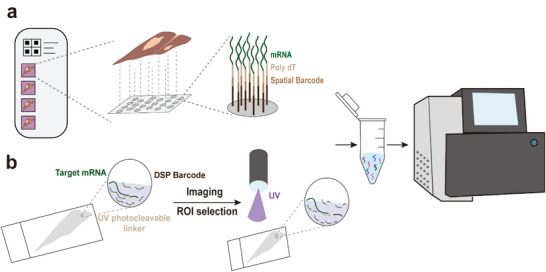
Commonly used spatially resolved transcriptomics technologies. a) 10x Visium. Tissue permeabilization releases mRNA to bind to the oligonucleotide array on slides. The cDNA obtained by reverse transcription is collected for library preparation and amplification. b) NanoString GeoMx. Special probes are bound to the target RNA, imaging and ROI selection are performed, and DNA indexed oligonucleotides are collected using UV photolysis. Finally, they are subjected to Illumina sequencing.

The Visium Spatial Gene Expression from 10x genomics, an optimization of spatial transcriptomics (ST),^[^ ^35^ ^]^ is a method that combines high‐resolution tissue imaging with high throughput spatially resolved RNA sequencing.^[^ ^36^ ^]^ It uses microarray slides distributed with spots 55 µm in diameter and 100 µm apart to capture RNA in tissues covered with slides. The spots contain barcodes with spatial location information, unique molecular identification tags, and poly(dT) oligonucleotide arrays (**Figure** **1**a). These spatial barcodes are unique to the corresponding spot to ensure that each captured molecule can be mapped to the original site using this barcode information, while poly(dT) probes are used to capture mRNA with poly(A) in permeabilized tissue slices. In simple terms, the tissue on the microarray slide is permeabilized to release mRNA for binding to the oligonucleotide array. The cDNA is synthesized on the slide by reverse transcription reactions, and the barcoded cDNA is pooled for downstream processing, library preparation, and amplification. After fragmentation and processing, transcriptome information is obtained by standard NGS short read long sequencing on an Illumina sequencer. Finally, the spatial barcodes are mapped back to each spot to achieve simultaneous acquisition of sequence information and positional information.^[^ ^37^ ^]^ Since then, another improvement in the technology has allowed it to break through the limitation of being applied only to fresh frozen tissue sections and thus support studies on formalin fixation and paraffin embedding (FFPE) tissues. For FFPE tissues, the tissue is de‐cross‐linked to release immobilized mRNA. A panel of human or mouse whole transcriptome probes is added to the tissue, which contains a pair of probes specific to each target gene. The target genes hybridize to the probe pair and are released by permeabilization of the tissue to bind to spatially barcoded oligonucleotides on the spot. The complement of spatial barcodes is added by an extension reaction and finally a library for sequencing is generated. The Visium Spatial Gene Expression for FFPE tissues enables FFPE tissue sections, which are widely used in scientific research, to complement traditional histopathological methods. Unfortunately, since 10x Visium operates on spots, each of which typically contains more than one cell, it has not yet reached single‐cell resolution and cannot yet provide deep transcriptome information on individual cells precisely in the tissue.

#### 2.2.2 NanoString GeoMx Digital Spatial Profiler

The GeoMx platform^[^ ^38^ ^]^ enables spatial in situ analysis of transcriptomes by combining microscopic imaging techniques and oligonucleotide tags^[^ ^39^ ^]^ and was launched in 2019 by NanoString. Samples are incubated for staining using reagents mixed with biological probes and fluorescent chromogenic markers, where each probe contains sequences complementary to the target RNA and DNA‐index oligonucleotides with ultraviolet (UV)‐cleavable linkers (Figure 1b), which are used to elucidate the morphology of the tissue. Reliance on fluorescent markers visually guide the manual selection of regions of interest (ROI) (60–800 µm), followed by photolysis using UV light in order to obtain the corresponding oligonucleotides by microcapillary aspiration.^[^ ^22^ ^]^ Finally, the collected oligonucleotides were counted or quantified using the nCounter system or the Illumina NGS platform^[^ ^40^ ^]^ to achieve accurate acquisition for spatial in situ gene expression profiles. This targeting approach quantifies transcripts in a way that can exclude biologically uninformative high expressions like ribosomal RNA.^[^ ^41^ ^]^ NanoString GeoMx also supports the analysis of proteomes with no damage to the sample throughout the process, which can be applied to precious samples. It is also extremely compatible in terms of what can be processed, allowing the manipulation of many types of samples such as fresh frozen tissue sections and FFPE sections, breaking the technical barrier that existed for years between unconventional samples and cutting‐edge technologies. However, similar to 10x Visium, GeoMx does not yet have true single‐cell resolution,^[^ ^42^ ^]^ as the acquired spatial gene expression is based on ROI rather than precise coordinates within the tissue,^[^ ^25^ ^]^ which includes at least 20 cells. The need to manually select the region also increases the difficulty of analyzing the entire tissue section, and the quality of fluorescence staining and the size of the selected ROI during the experiment will have a significant impact on the final result. In addition, GeoMx is less flexible and is clearly more suitable for questioning rare cell populations or providing precise information of greater interest to the researcher than 10x Visium.^[^ ^25^ ^]^

In addition to the above two, several other NGS‐based SRT technologies with respective characteristics in terms of tissue integrity (destructive or non‐destructive), resolution (multicellular, cellular, or subcellular), and field of view (large or small)^[^ ^43^ ^]^ have been developed and have found initial applications in different biological fields. Inspired by optical tomography techniques such as computed tomography, Tomo‐seq^[^ ^44^ ^]^ combines traditional histological techniques with low‐input RNA sequencing and digital image reconstruction for the generation of 3D spatially resolved transcriptomics profiles, especially in the field of developmental biology. This technology has certain advantages in terms of RNA quantification and spatial resolution, but “artifacts” may appear during image reconstruction and it cannot be applied to clinical samples of tumor.^[^ ^19^ ^]^ LCM‐seq^[^ ^45^ ^]^ is a technology that combines laser capture micro‐cleavage (LCM) and the Smart‐seq2 for accurate SRT analysis. It is easier, less costly, and more sensitive to preserve the positional information of cells without separating tissues, while acquiring transcriptome profiles of rare cell populations in known locations when fewer cells are required. Geo‐seq^[^ ^46^ ^]^ is similar in principle to LCM‐seq, and has almost no restriction for sample types, but the relatively low throughput is one of its drawbacks. HDST is another improved version of the ST,^[^ ^47^ ^]^ where magnetic beads replace spot arrays and are able to measure in situ tissue spatial information at a resolution 1400 times higher than the ST. Even though HDST has a high resolution and signal specificity, the data obtained are relatively sparse, and higher resolution also implies greater noise.^[^ ^48^ ^]^ Slide‐seq^[^ ^49^ ^]^ is a scalable technique for large tissue sizes,^[^ ^50^ ^]^ which creates spatial indexes by using 10µm diameter DNA barcode beads called “beads”.^[^ ^51^ ^]^ It has achieved a breakthrough in single‐cell resolution, but the beads get transcriptional profiles from both cells if they are located at the boundary between the two cells.^[^ ^52^ ^]^ In addition, the low sensitivity of transcript detection, small number of detection genes, incompatibility with histological images,^[^ ^53^ ^]^ high experimental technique requirements, and inapplicability to fixed tissues have limited the widespread use of this technology. On the other hand, Slide‐seqV2^[^ ^54^ ^]^ has improved library preparation, beads synthesis and array index on the basis of slide‐seq, resulting in a nearly 10‐fold increase in RNA capture efficiency and greatly expanding the range of possible analyses. Sci‐Space^[^ ^55^ ^]^ achieves single‐cell resolution by labeling cell nuclei in combination with RNA sequencing, but with a lower spatial resolution (200 µm) and a smaller number of transcripts obtained. After two rounds of sequencing, Seq‐Scope^[^ ^56^ ^]^ has the advantages of high output resolution, high number of transcriptome captures, scalability, and adaptability. DBiT‐seq^[^ ^33^ ^]^ is a completely different approach, which does not rely on solid‐phase matrix capture of transcripts, but obtains positional information by direct spatial encoding. Its resolution depends on the channel width, and although the highest resolution is close to the single‐cell level, it cannot be directly applied to resolve single cells. DBiT‐seq has been shown to be applicable to the richest archival specimens of FFPE tissue, but its performance needs to be further improved. In addition, the pixel size and the limited number of channels in the chip are also technical challenges that have not yet been solved.^[^ ^57^ ^]^ Stereo‐seq^[^ ^58^ ^]^ combines DNA nanoball (DNB) pattern arrays and in situ RNA capture technology to resolve SRT. The standard DNB chip has a spot diameter of ≈220 nm and a spot‐to‐spot distance of 500 or 715 nm. Compared with other SRT technologies, Stereo‐seq has a larger number of spots in the same area and a smaller spot size and spot‐spot‐center distance, and thus features a large field of view, nanoscale resolution, high sensitivity, and uniform capture rate. Light‐Seq^[^ ^59^ ^]^ is a direct integration of microscopy and NGS to obtain spatial indexes using light‐guided DNA barcodes, which offers great advantages in terms of sensitivity, spatial resolution, and ease of adoption in combination, but the number of detectable regions is yet to be further increased. Currently, SRT technologies are rapidly evolving and are being quickly developed and improved, but it is important to keep in mind that the development of new technologies is not the ultimate goal. It is more important to apply them in practical studies to provide us with more insights into the inner workings of the tumor. **Table** **2** shows the significant achievements of the application of SRT in cancer research.

**Table 2:** Overview of cancer research using SRT technology.

Cancer Type	SRT Technology	Highlights	References
Prostate cancer	ST	Histopathology corresponds to gene expression patterns	[61]
		Differential gene expression exists between the core and periphery of cancer	
Triple‐negative breast cancer	10x Visium	Triple‐negative breast cancers of different races have a conserved spatial transcriptional structure	[63]
		Hypoxia may significantly affect the tumor environment in African American triple‐negative breast cancer	
Prostate cancer	10x Visium	Spatially resolved transcriptional profiles can be used to infer spatial copy number variations	[66]
Breast cancer	ST	High heterogeneity even in areas with the same ductal carcinoma in situ	[35]
Breast cancer	Public datasets	Applying machine learning to breast cancer tissue region classification	[70]
Cutaneous malignant melanoma	ST	Cancer regions in long‐term survivors exhibit more uniform gene expression patterns compared to short‐term survival patients	[71]
		The expression pattern of lymphoid tissue regions may depend on their distance from tumor cell clusters	
Invasive micropapillary carcinoma of the breast	10x Visium	Metabolic reprogramming is an important feature of the Invasive micropapillary carcinoma region	[73]
		Lack of antitumor effect of lymphocyte infiltration in the region of Invasive micropapillary carcinoma	
		Gene expression characteristics of stromal regions are influenced by their distance from Invasive micropapillary carcinoma	
Lung adenocarcinoma	10x Visium	UBE2C^+^ cancer cell subpopulation contributes to the invasive process of lung adenocarcinoma	[74]
		Treg increase and monocyte decrease promote early lung adenocarcinoma invasion	
		Malignant features of tumor margins become more active in lung adenocarcinoma progression	
Hepatocellular carcinoma	10x Visium	Two distinct gene profiles associated with satellite nodules were identified	[75]
		A prognostic model consisting of six sub‐cluster‐specific marker genes was constructed	
Colorectal cancer	10x Visium	Several genes, including AKR1B1, are associated with colorectal cancer invasion	[76]
Breast cancer	10x Visium	Disseminated cancer cells with high levels of oxidative phosphorylation are distributed in clusters at the tumor front as an early event of lymph node metastasis	[77]
Pancreatic ductal adenocarcinomas	ST	Activated fibroblasts are enriched in the cancer region rather than the stroma region	[83]
		Different macrophage subpopulations are enriched in different regions of the tissue	
Colorectal cancer	10x Visium	FAP^+^ fibroblasts co‐localize spatially with SPP1^+^ macrophages, inhibiting immune cell infiltration and reducing the effect of immunotherapy	[84]
Breast cancer	10x Visium	CAFs are divided into three superclusters: steady state‐like, mechanoresponsive, and immunomodulatory	[86]
Cervical squamous cell carcinoma	Stereo‐seq	Cancer‐associated myofibroblasts support tumor cell growth and metastasis by inhibiting lymphocyte infiltration and tumor extracellular matrix remodeling and are associated with poor prognosis	[87]
Squamous cell carcinoma	ST	The tumor‐specific keratinocyte subpopulation is localized at the tumor front and interacts differently with various types of neighboring cells	[7]
Basal cell carcinoma	NanoString GeoMx	Basal carcinoma cells strongly express the cytokine Activin A and act in the adjacent stromal region, mediating fibroblasts to support their formation of an invasive ecological niche	[88]
Ovarian cancer	10x Visium	CAF‐specific subtypes and their relative spatial location to specific ovarian cancer cell subpopulations affect patient survival time	[89]
Prostate cancer	Slide‐seqV2	“Prostate Tumor Gene Signature” can robustly identify tumor samples	[90]
		Increased abundance of immunosuppressive myeloid subpopulations, T‐cell depletion and immunosuppressive Treg activity are characteristic of prostate cancer	
Liver cancer	10x Visium	Tumor capsule affects intra‐tumor spatial continuity, transcriptional diversity, and immune cell distribution	[95]
		PROM1^+^ cancer stem cell plays an important role in tumor progression	
		“TLS‐50” can identify TLS with high confidence	
Colorectal cancer	10x Visium	Preferential reprogramming of macrophages and induction of their specific functional state is the difference between primary and hepatic metastatic cancer cells	[9]
		Dramatic increase in metabolic activity of MRC1^+^CCL18^+^ macrophages in liver metastasis samples	
		Neoadjuvant chemotherapy reprograms the intra‐tumor immune balance and activates the systemic anti‐tumor response	
Breast cancer	10x Visium	The spatial distribution of macrophages with both M1 and M2 characteristics is closely related to tumor cells	[98]
		CAFs and macrophages are involved in forming the immunosuppressive microenvironment, with the former in particular making the greatest contribution	
Non‐small cell lung cancer	NanoString GeoMx	Patients with CD163^+^ cells in tumors that are more distant from tumor cells and have lower levels of infiltration have longer survival	[99]
Colorectal cancer	10x Visium	Aggressive frontier colorectal tumor cells suppress tumor immunity by converting macrophages to SPP1^+^ macrophages through secretion of HLA‐G	[100]
Hepatocellular carcinoma	10x Visium	CCL15 recruits monocytes and polarizes them into M2‐type macrophages, promoting the formation of an immunosuppressive microenvironment	[101]
		CCL19 and CCL21 can enrich T cells and B cells and inhibit cancer cell growth	
Hepatocellular carcinoma	10x Visium	SPP1^+^ macrophages interact with CAFs to form a tumor immune barrier and limit immune infiltration	[102]
Hepatocellular carcinoma	10x Visium	Production of TGF‐β by M2‐type macrophages and CAFs in steatotic hepatocellular carcinoma promotes depletion of peripheral CD8^+^ T cells	[103]
Breast cancer	ST	CXCL10^+^ Mø2 co‐localizes spatially with IFIT1^+^ T cells, indicating the recruitment of the former to the latter	[108]
		“TLS signature” has the ability to identify tertiary lymphoid‐like structures	
Renal cell cancer	10x Visium	“TLS imprint signature” is used to determine the spatial location of TLS in the tumor	[109]
		B cells expand and mature into plasma cells in TLS and migrate to tumor lesions guided by CXCL12^+^ fibroblasts, while secreting IgG and IgA antibodies against tumor cells	

CAF: Cancer‐associated fibroblast; TLS: Tertiary lymphoid structures.

## 3 Applications of SRT Technology in Cancer Research

### 3.1 SRT Technology in Revealing Tumor Cell Heterogeneity

Tumor cells are the central objects of tumor research, which originate from normal cells that undergo oncogenic transformations.^[^ ^60^ ^]^ The detection and destruction of these malignant cells in the early stages of cancer is one of the ultimate goals of anticancer therapy. SRT technology is excellent in discriminating tumor cells from normal cells differentially.

As a result of genetic mutations, the transcriptional profile of tumor cells also changes, which can serve as a cue for early stages of cancer. A factor analysis method based on the unsupervised probability framework was able to identify histological spatial gene patterns, as demonstrated by the ability to predict normal gland, stroma, and cancerous or prostatic intraepithelial neoplasia (PIN) regions in prostate cancer tissue sections. The accuracy of this method was also validated by traditional hierarchical clustering and principal component analysis (PCA) methods, confirming the association between histological entities and gene expression profiles. Cancer regions and PIN regions also have region‐specific markers, for example, genes SPINK1 and PGC are enriched in the former and NPY is highly expressed in the latter.^[^ ^61^ ^]^ From a clinical point of view, pathologists can refer to the results of this analysis to give special attention to regions of high expression of cancer‐related genes in order to determine the extent of the lesion at an early stage before any histological changes are present. The ESTIMATE^[^ ^62^ ^]^ method can score tumor purity by inferring the ratio of stromal cells and immune cells in triple‐negative breast cancer samples to accurately identify tumor regions.^[^ ^63^ ^]^ An integrated tool framework Cottrazm^[^ ^64^ ^]^ has an excellent performance in delineating core tumor regions based on SRT and scRNA‐seq data. Genetically related cell populations that accumulate through heritable mutations become distinct populations, referring as clones.^[^ ^65^ ^]^ Genome sequencing is the most direct way to analyze the evolution of cancer clones, but it is a huge task. Methods such as inferCNV and CopyKAT have been used to infer copy number variations (CNVs) and clonal structures from RNA sequencing data.^[^ ^22^ ^]^ It has been shown^[^ ^66^ ^]^ that SRT has become a useful tool for genome‐wide analysis and that spatially resolved mRNA profiles are reliable tools to infer spatially resolved genome‐wide CNV in benign and malignant tissues, revealing their respective distinct clonal patterns. Even within the same pathological partition, the variability in gene expression suggests the presence of different subclones.^[^ ^35^ ^]^ This may be instructive for gaining insight into the structure, nature, and evolution of tumor clones and for improving the early diagnosis of cancer.

Ductal cancer in situ (DCIS), is a precursor of invasive ductal cancer (IDC) with low malignancy, allowing for less aggressive treatment.^[^ ^67^ ^]^ Therefore, personalized local treatment targeting only the more malignant IDC areas becomes a fundamental requirement to avoid overtreatment and excessive costs in breast cancer.^[^ ^68^ ^]^ However, fully differentiating between DCIS and IDC is a challenging task for pathologists.^[^ ^69^ ^]^ On morphology identified breast cancer biopsy sections with six separate DCIS regions and one IDC region, and STÅHL et al.^[^ ^35^ ^]^ found higher expression of extracellular matrix‐related genes such as VIM and FN1 in the latter by SRT analysis, suggesting their important role in DCIS infiltration transformation and that they could be used as signature genes to distinguish DCIS from IDC. Niyaz et al.^[^ ^70^ ^]^ trained and tested a machine learning method that automatically selected cell types based on pathologist annotations and transcriptome profiles, which could help pathologists with clinical decision support by learning cancer features from expert annotated SRT data for DCIS and IDC classification in breast tissue sections. In addition, Thrane et al.^[^ ^71^ ^]^ used the same factor analysis method^[^ ^68^ ^]^ to identify two different tumor profiles in the SRT data of four stage III skin malignant melanoma samples with lymph node metastases. Melanoma‐A factors, including CD63 and PMEL, were generally highly expressed within the melanoma region of patient 1, whereas enhanced expression of melanoma‐B, such as S100B and FTH1, was evident in patient 4 and was not observed in patient 1. All of the above suggest that SRT analysis can yield a more complex transcriptional landscape than that obtained by histopathological analysis to help identify or confirm new clinicopathological typing and enable precise treatment by specifically targeting different subtypes.

Metastasis is closely related to treatment resistance and is the leading cause of death in most cancer patients. Some genes promote tumor metastasis and can be serve as potential therapeutic targets.^[^ ^72^ ^]^ Invasive micropapillary carcinoma (IMPC) is a specific histological subtype of breast cancer that is more prone to lymphatic vessel invasion and lymph node metastasis than IDC and has a worse prognosis. Lv et al.^[^ ^73^ ^]^ first mapped the spatially resolved transcriptome of IMPC and found that SREBF1/FASN was closely associated with IMPC invasion and metastasis. An epithelial cell subpopulation UBE2C^+^ Epi‐C6 identified by scRNA‐seq data had a much smaller spatial distribution ratio in situ lung adenocarcinoma (LUAD) than in microinvasive adenocarcinoma. And ssGSEA enrichment analysis showed that this subpopulation was more enriched in MYC target V1, cell cycle, AKT, and TGF‐β signaling pathways, indicating that they contribute to LUAD invasion and metastasis through these pathways. In addition, more UBE2C^+^ Epi‐C6 was distributed in the peripheral cancer region compared to the central cancer region, showing a higher oncogenic profile, demonstrating that UBE2C is the driver gene of LUAD metastasis.^[^ ^74^ ^]^ Microsatellite nodules of hepatocellular carcinoma (HCC) are small cancerous foci that appear around the liver tissue of the primary tumor. More tumor clusters were identified in tissues with microsatellite nodules compared to tissues without microsatellite nodules,^[^ ^75^ ^]^ in which NDRG1, BHLHE40, and VEGFA genes related to invasion and metastasis, were highly expressed and could serve as new markers for microsatellite nodules. By inferring the differentiation trajectory of colorectal cancer (CRC) cell invasion through pseudo‐time and cell experiments, Liu et al.^[^ ^76^ ^]^ proposed that AKR1B1 promotes the proliferation, invasion, and migration of CRC cells. Early disseminated cancer cells clusters were located at the front of the tumor region and showed a significant enrichment of the OXPHOS pathway compared to normal breast ductal epithelial cells. Further analysis and experiments showed that the marker genes COX6C and DHRS2 in OXPHOS promote the proliferation and migration of breast cancer cells as knocking them down inhibits invasion.^[^ ^77^ ^]^ Overall, the identification of metastasis‐promoting genes by SRT data is beneficial for preventing cancer spread and treating metastasis.

### 3.2 SRT Technology in Studying Stromal Cells in TME

Stromal cells in the tumor microenvironment include but are not limited to vascular endothelial cells, lymphatic endothelial cells, mesenchymal stem cells, and cancer‐associated fibroblasts (CAFs). They can promote tumor cell growth, proliferation, and metastasis by secreting growth factors required by tumor cells, supporting angiogenesis, inducing epithelial mesenchymal transformation in tumor cells, and constructing physical immune barriers.^[^ ^78^ ^]^ Based on their tumor‐supporting effects, it is believed that targeting stromal cells to reduce tumor burden is feasible.

As the most abundant stromal cell in the tumor microenvironment, CAFs are the most attractive treatment targets for many solid tumors.^[^ ^79^ ^]^ However, no successful clinical therapies targeting CAFs have been developed to date,^[^ ^80^ ^]^ partly because the heterogeneity and functions of CAFs are not yet well understood.^[^ ^81^ ^]^ CAFs are highly diverse in origin, subpopulation character, and function, and even though they have also been claimed to have cancer suppressive effects in some cases, more current evidence suggests that they promote tumors.^[^ ^82^ ^]^

**Figure 2:**
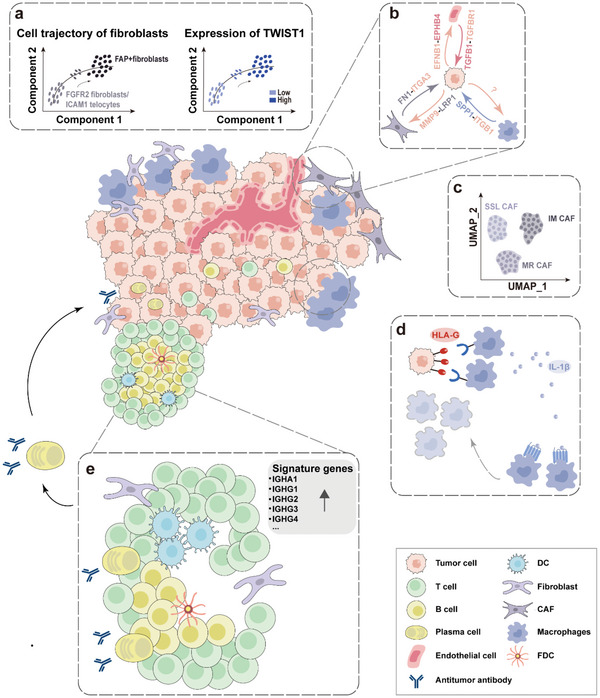
Spatial mapping of solid tumors using SRT technology. a) FAP^+^ fibroblasts are obviously enriched in CRC tissues. Cell trajectory analysis infers that they originate from FGFR2^+^ fibroblasts or ICAM1^+^ terminal cells, and this process is driven by the transcription factor TWIST1. The enrichment of FAP^+^ fibroblasts and SPP1^+^ macrophages form a physical barrier to restrict the infiltration of immune cells into the tumor, leading to an immune‐suppressive CRC microenvironment. b) Invasive epithelial subsets of TSK have complex interactions with CAFs and endothelial cells at the tumor‐stromal interface through multiple receptor complexes. c) CAFs are innovatively described as three superclusters: steady‐state‐like (SSL), mechanoresponsive (MR), and immunomodulatory (IM) CAFs. d) CRC cells at the invasion front express high levels of HLA‐G to induce an increase in of SPP1^+^ macrophages, which secrete IL‐1β to recruit additional SPP1^+^ macrophages, and they together deplete other immune cells and suppress tumor immunity. e) B cells expand and mature into plasma cells within the TLS and exhibit high secretion of IgG and IgA antibodies targeting tumor cells. Feature gene sets of TLS are highly expressed in this region.

Multimodal intersection analysis (MIA)^[^ ^83^ ^]^ is a method to integrate SRT and scRNA‐seq data by calculating the degree of overlap between cell type‐specific and region‐specific gene sets. Based on MIA, Moncada et al. found that fibroblasts from pancreatic ductal adenocarcinoma (PDAC) were enriched in the cancer region rather than the stroma region, indicating that they are activated fibroblasts, and it is known that only activated fibroblasts promote tumorigenesis and progression. A separate scRNA‐seq combined with SRT analysis study^[^ ^84^ ^]^ showed that FAP^+^ fibroblasts were significantly enriched in CRC tissues but not adjacent normal tissues. The differentiation trajectory of FAP^+^ fibroblasts was inferred by RNA velocity algorithm, and the results showed that they may originate from FGFR2^+^ fibroblasts or ICAM1^+^ terminal cells, and pySCENIC analysis concluded that this differentiation was mainly driven by the transcription factor TWIST1 (**Figure** **2**a). In previous studies, CAFs were generally divided into two subpopulations, myofibroblastic CAFs (myCAFs) and inflammatory CAFs (iCAFs).^[^ ^85^ ^]^ Foster et al.^[^ ^86^ ^]^ used scRNA‐seq, SRT, and spatial proteomics to study CAFs and innovatively described them as three superclusters: steady‐state‐like (SSL), mechanoresponsive (MR), and immunomodulatory (IM) CAFs (Figure 2c), which expanded the biological understanding of CAF subpopulations.

CAFs have generally been shown to be beneficial to tumor cells. Ou et al.^[^ ^87^ ^]^ identified a myCAF cluster through combination of single nuclear RNA sequencing and Stereo‐seq data from cervical squamous cell carcinoma (CSCC). It promotes the malignant progression by up‐regulating key tumorigenic pathways, acting as a physical barrier to immune cell infiltration, reconstructing extracellular matrix, and overexpressing secretory factors to enhance cancer cell stemness and proliferation, demonstrating that intervention against myCAFs could be complementary to current CSCC therapies. Another study^[^ ^7^ ^]^ identified a specific subpopulation of epithelial cells with invasive behavior and high cellular state plasticity in cutaneous squamous cell carcinoma, which were called tumor‐specific keratinocyte (TSK). More than 80% of TSKs were located at the anterior margin of the tumor, and CAF and endothelium‐related transcripts were highly enriched in the adjacent region, indicating their contribution to the fibrovascular niches surrounding TSK cells. Furthermore, based on the ligand‐receptor database, NicheNet analysis revealed that TSK mediates signaling to CAF through ligand‐receptor pairs such as MMP9‐LRP1 and TNC‐SDC1, as well as regulates endothelium through PGF‐FLT1, PGF‐NRP2, and EFNB1‐ EPHB4. In turn, CAFs and endothelial cells co‐express ligands TFPI, FN1, and THBS1 to activate TSK receptors, further demonstrating complex interactions between CAFs and endothelial cells and TSK (Figure 2b). A study^[^ ^88^ ^]^ of the invasive interface of basal cell carcinoma showed increased expression of cell‐cell junction complexes in tumor cells, which exhibit a collective migration phenotype. At the same time, these tumor cells strongly expressed the cytokine Activin A, and the gene markers induced by Activin A were also increased in CAFs co‐located with the tumor cells, indicating that CAFs support the formation of an aggressive niche of tumor cells. The association between CAFs and survival in patients with advanced high‐grade serous ovarian cancer(HGSC) has attracted the attention of Ferri‐Borgogno et al.^[^ ^89^ ^]^ Ligand‐receptor analysis at the mesenchymal‐tumor interface showed that APOE (ligand expressed on CAFs)‐LRP5 (receptor expressed on tumor cells) was present in both short‐term survival patient samples, and subsequent cell experiments also demonstrated the effect of this crosstalk network on HGSC cell survival. Hirz et al.^[^ ^90^ ^]^ attempted to infer ligand‐receptor interactions in physically adjacent cells from Slide‐seqV2 data, and found that COL3A9‐ITGA2 is present in tumor cells with fibroblasts, and is involved in extracellular matrix remodeling and cell migration. Blocking these interactions may be useful in limiting the supporting effects of CAFs on tumor cells and slowing down cancer progression. SRT technology has helped researchers to take a deep look at the spatially specific distribution of CAFs, not just all CAFs in a broad sense but also different subpopulations, deepening our understanding of the link between the spatial location of CAFs and their functions.

### 3.3 SRT Technology in Exploring the Tumor Immune Microenvironment

With the success of tumor immunotherapy, researchers have gradually focused their attention on immune cells. Innate and adaptive immune cells such as myeloid cells and lymphocytes constitute the majority of tumor immune microenvironment.^[^ ^91^ ^]^ In patients, antagonistic tumor immune cells are suppressed, tumor‐promoting immune cells are prevalent.^[^ ^92^ ^]^ Tumor cells, stromal cells, and these immune cells interact and influence each other to dynamically regulate the tumor progression, which is a key factor preventing the complete tumor cure.

The ability to understand the spatial distribution of various types of immune cells in the tumor microenvironment is an advantage of SRT. Macrophages are highly plastic and can adjust their identity and function in response to external stimuli.^[^ ^93^ ^]^ At the same time, they are the most abundant innate immune cell population in tumors, representing an important therapeutic target.^[^ ^94^ ^]^ MIA^[^ ^83^ ^]^ analysis revealed that two subpopulations of macrophages, the pro‐inflammatory and anti‐tumor M1 type and the anti‐inflammatory and pro‐tumor M2 type have opposite enrichment patterns in PDAC patients, reflecting macrophage function in tissue‐resident and inflammatory environments, respectively, as they are enriched in stromal and cancerous areas as well as in the ducts.^[^ ^83^ ^]^ The MIA mapping provides evidence that these different subpopulations play different roles in different parts of the tissue. HCC tissues with fibrous capsule have higher spatial continuity and lower transcriptional diversity, with further studies indicating that the presence of fibrous capsule affects the distribution of immune cells such as T, B, and myeloid cells in HCC.^[^ ^95^ ^]^ Myeloid‐derived suppressor cells are the most important protective cells against tumor cell destruction.^[^ ^96^ ^]^ They have been found to have increased infiltration activity in tumor, similar to Treg, which is important for the formation of immunosuppressive microenvironment in human prostate cancer, as well as the depletion of T and NK cells.^[^ ^90^ ^]^ Increased Tregs and decreased monocytes have been shown to activate tumor‐associated pathways, thereby promoting early LUAD invasion.^[^ ^74^ ^]^ Liver is the most important target organ for CRC metastasis, and colorectal cancer liver metastasis (CRLM) has a highly heterogeneous and suppressive immune microenvironment, which is one of the bottlenecks and difficulties for CRC treatment. By scRNA‐seq and SRT sequencing of paired samples of CRC and liver metastasis, Wu et al.^[^ ^9^ ^]^ found that immunosuppressive cells such as SPP1^+^ macrophages and MRC1^+^CCL18^+^ macrophages were specifically enriched in liver metastasis samples and which was validated in open independent cohorts, revealing the role of these macrophage subpopulations in the tumor‐primary ecological niche of CRLM formation. Effective neoadjuvant chemotherapy (NAC) can modulate the levels of these macrophage subpopulations as well as cytotoxic immune cells, reprograming the immune homeostasis within the tumor, suggesting a potential role for NAC in resectable CRLM. The use of SRT to understand the distribution levels of these immune cells is necessary because “cold tumors” and “hot tumors” respond differently to immunotherapy.^[^ ^97^ ^]^

It is well known that tumor cells and immune cells influence each other's fate in a bidirectional manner. Regions of the lymphatic system adjacent to and distant from the melanoma region were grouped into two separate PCA clusters based on gene expression profiles. Factor analysis^[^ ^61^ ^]^ revealed the involvement of IGLL5 and CD74 in this cluster separation and their specific presence in both regions, suggesting that gene expression program in the lymphoid tissue is influenced by the distance of tumor cells.^[^ ^71^ ^]^ TGF‐β signaling between tumor cells and TME cells, especially NK, mast, and MALT B cells, plays a key role in the invasion of advanced LUAD.^[^ ^74^ ^]^ One macrophage subpopulation, Mac.CCL3.4, expressed high M1 and low M2 characteristics, while another, Mac.FABP5, expressed high M1 and M2 characteristics. More importantly, Mac.FABP5 showed higher spatial co‐localization with tumor cells than Mac. CCL3.4, demonstrating the important influence of malignant environment on macrophage polarization.^[^ ^98^ ^]^ In a study by Larroquette et al.^[^ ^99^ ^]^ applying Nanostring GeoMx to the level of macrophage infiltration in non‐small cell lung cancer, the results showed that the distance between tumor‐associated macrophages (TAMs) from tumor cells directly affected patient prognosis, rather than the infiltration level in the stroma. Patients whose TAMs were farther away from the tumor cells had a longer survival. Tumor cells at the invasion front induce the increase of SPP1^+^ macrophages by expressing high levels of HLA‐G, and SPP1^+^ macrophages secrete IL‐1β to recruit additional SPP1^+^ macrophages (Figure 2d), and together they inhibit anti‐tumor immunity by depleting other immune cells. It provides new theoretical support for improving the prognosis of patients with CRC.^[^ ^100^ ^]^ Wang et al.^[^ ^101^ ^]^ used pseudo‐time analysis to reconstruct the trajectory of SRT spots from HCC, and observed that the oncogenic chemokine CCL15 gradually accumulated along the pseudo‐time trajectory and dominated at the end. Further experimental results showed that CCL15 was able to recruit monocytes and polarize them into M2‐like macrophages to promote the formation of immunosuppressive microenvironment in HCC. On the contrary, chemokines CCL19 and CCL21 enriched T and B cells and inhibited HCC growth. Altogether, SRT analysis revealed complex spatial distribution patterns in the HCC immune microenvironment, providing new insights for the development of precise therapeutic strategies for HCC.

In addition to tumor cells, the immune microenvironment is also influenced by stromal cells, especially CAFs, which exert immunosuppressive functions by interacting with immune cells.^[^ ^91^ ^]^ It has been shown that SMA^+^VIM^+^PDGFRB^+^ CAFs form an immune barrier, thus preventing CD4^+^ central memory, CD8^+^ effecting memory, monocytes, NK, B, myeloid dendritic cells, and plasmacytoid dendritic cells from into the tumor, leading to short‐term survival patients.^[^ ^89^ ^]^ Similar to fibroblast analysis process,^[^ ^84^ ^]^ it was found that SPP1^+^ macrophages are tumor‐specific macrophages and may originate from THBS1^+^ macrophages. Further, the authors found that FAP^+^ fibroblasts and SPP1^+^ macrophages co‐localized spatially (Figure 2a), and promoted connective tissue proliferation through complex interactions such as reciprocal recruitment and activation to limit the infiltration of immune cells into the tumor interior, leading to further deterioration of CRC microenvironment and ultimately to a reduction in the efficacy of PD‐L1 therapy. These results highlight that targeting FAP^+^ fibroblasts, SPP1^+^ macrophages, and molecules involved in their interactions may increase tumor response to immunotherapy. Similar interactions between SPP1^+^ macrophages and CAFs have also been reported in liver cancer. By comparing the difference in the ratio of each cell subpopulation between HCC and adjacent normal tissues, Liu et al.^[^ ^102^ ^]^ found that SPP1^+^ macrophages and CAFs were significantly increased in HCC. Cell communication analysis also showed significant interactions between the two, which could prevent infiltration of CD8^+^ T cells into the tumor center by forming a barrier. Targeting SPP1 will increase the efficacy of anti‐PD‐1, which could be a potential target for enhanced immunotherapy. A study by Mao and colleagues^[^ ^98^ ^]^ on breast cancer found that immunodeficient cell populations such as Treg and CAF have lower tumor space specificity, suggesting that they may build an immunosuppressive microenvironment not by interacting with themselves but with other immune cells. Further studies on how to block such interactions may provide new ideas for breast cancer treatment. Immune checkpoint blockade therapies especially with PD‐L1/PD‐1 and CTLA‐4 inhibitors have significantly improved the treatment efficacy for many malignancies, but not all patients have benefited and new checkpoints need to be looked into. Resolution of the immune microenvironment of CSCC observed significantly higher levels of LGALS9 and IDO1 than non‐cancer samples. LGALS9 and IDO1 may serve as targets for improving CSCC therapy, but the impact of myCAFs in this should be considered, as it limits lymphocyte infiltration.^[^ ^87^ ^]^ To investigate the immune‐depleted tumor immune microenvironment in steatotic HCC, Murai et al.^[^ ^103^ ^]^ defined CD8A^+^NR4A1^+^ spots as depleted cytotoxic T‐lymphocytes (CTLs). By comparing their transcriptional profiles with those of non‐depleted spots, increased expression of the M2 macrophage marker CD163 and the CAF marker VIM was found in them, suggesting that the immune depletion microenvironment in steatotic HCC may be caused by M2 macrophages and CAFs promoting the depletion of surrounding CTLs. Exploring the co‐localization and interaction of CAF‐immune cell could serve as a basis for establishing new oncology drug targets and biomarker libraries.

### 3.4 SRT Technology in Identifying Tumor‐Associated TLS

In addition to the study of the phenotypes, functions, and interactions of various cells, SRT is uniquely positioned to explore the specific spatial structures in the TME. TLS is an ectopic lymphoid structure developed in tumor areas or chronic inflammatory areas. It is a cellular aggregate composed mainly of B and T cells and has been identified in a variety of cancer types.^[^ ^104^ ^]^ TLS in the tumor microenvironment can promote the infiltration of immune cells into the tumor site, thereby enhancing the patient's systemic persistent antitumor immune response. Therefore, its presence is often considered to be associated with good prognosis in most solid tumors,^[^ ^105^ ^]^ which has aroused a strong interest in studying TLS. Multiple immunohistochemistry (CD20, CD3, CD8, PNAd, or DC‐LAM)^[^ ^106^ ^]^ or multiple immunofluorescence is usually used to identify and locate TLS, but this is a highly difficult task. At present, the results of several studies have shown that SRT technology is helpful to study the cellular composition of TLS regions and their distribution in space, so as to provide preliminary ideas for the formation of reproducible and robust TLS for tumor treatment.

Although the co‐localization of B and T cells does not fully indicate the presence of TLS, it can be used as a hint since these two cells constitute the majority of immune cells associated with TLS.^[^ ^107^ ^]^ The score for the joint presence of these two cells, defined as the TLS score, was used by Andersson et al.^[^ ^108^ ^]^ to discover TL‐like structures that are part of the constitutive TLS. Based on the relationship between TLS score and gene expression, a linear model that can be widely generalized to predict TL‐like structure was constructed, and the gene with the highest contribution to positive score was called “TLS signature”. These TLS signatures were found to be highly correlated with cell activation, differentiation, and immune response or regulation by functional enrichment analysis. Similarly, another study also proposed the concept of “TLS‐50”, which was used to examine the presence and distribution of TLS region with high confidence with the help of SRT. It was found that TLS predominantly present in L slices with both tumor and paracancerous areas, but not in tumor slices or non‐tumor slices. At the same time, there is also a potential correlation between the proportion of cells constituting TLS and their distance to the tumor area, indicating the influence of tumor cells on composition of TLS.^[^ ^95^ ^]^ The first study to apply 10x Visium to FFPE samples^[^ ^109^, ^110^ ^]^ revealed the presence of tightly clustered B cell and T cell regions within the TLS region associated with renal cell carcinoma identified by pathologists, and these regions could be visualized in SRT data using a “TLS imprint signature” composed of 29 genes. Subsequent analysis showed that B cells expanded and matured into plasma cells within the TLS, migrated to tumor foci under the guidance of CXCL12^+^ fibroblasts, and exhibited high secretion of IgG and IgA antibodies targeting tumor cells (Figure 2e), thereby promoting antitumor effects. This suggests that induction of B cells expansion and maturation and IgG antibody production in TLS could be a novel tumor therapy. These evidences also show that identifying and locating TLS structures is one of the best applications to demonstrate the advantages of SRT technology, as it does not destroy the original tissue structure. Therefore, it is necessary to take full advantage of these TLS‐associated signature gene sets (**Table** **3**) to robustly identify and localize TLS at the transcriptome level to reduce the workload of pathologists.

**Table 3:** Feature gene sets for identifying TLS in SRT data.

Feature Gene Sets	Signature Genes	Cancer Type	SRT Technology	References
TLS‐50	FDCSP, CR2, CXCL13, LTF, CD52, MS4A1, CCL19, LINC00926, LTB, CORO1A, CD79B, TXNIP, CD19, LIMD2, CD37, ARHGAP45, BLK, TMC8, CCL21, PTPN6, ATP2A3, IGHM, SPIB, TMSB4X, CXCR4, NCF1, CD79A, ARHGAP9, DEF6, EVL, TBC1D10C, RASAL3, INPP5D, RNASET2, RASGRP2, TNFRSF13C, RAC2, CD22, ARHGEF1, AC103591.3, TRAF3IP3, HLA‐DQB1, CD53, ARHGAP4, TRBC2, POU2AF1, TRAF5, OGA, FCRL3, HLA‐DQA1(50)	Liver cancer	10x Visium	[95]
TLS‐signature	MS4A1, B2M, TRBC2, LTB, TIAM1, ZC3H12D, SCML4, CXCR4, CD19, TRAF3IP3, BTG1, BIRC3, CD52, RAC2, CD83, HLA‐B, PTPRC, TRAC, SLA, CXCL9, CORO1A, IL16, CYTH1, ZFP36L2, ITGAL, ICOS, FAM96B, CXCL13, PASK, CCR7, SNX13, TRAF5, LRMP, BLK, IL7R, PRKCB, IL11RA, IL2RG, CD247, QRICH1, CARMIL2, C5orf56, CD3E, DNMBP, RHOF, ARHGEF1, KLF12, TXNIP, LYZ, TCL1A, SAFB, DGKA, BRE, PLEKHG1, PTPN22, LAMP5, ZNF558, JAK3, CD69, SYT11, FYB, CD48, PLA2G2D, SPIB, LIMD2, ARHGAP15, BCL11B, AKNA, TNFAIP8, CD2, DDX5, POGLUT1, TIPARP, GAREM2, EPHA4, IL2RB, GRPEL2, ADAM28, VWA8, FAR1, TMSB4X, CACNA1I, SMCHD1, HLA‐DQB1, LNPEP, RCHY1, GPSM3, TRBC1, TSC22D3, DOCK8, SPOCK2, PIP4K2A, CCDC50, GZMK, CPSF7, CD53, SLAMF6, BARX2, FUCA1, CCL19, ACAP1, IBTK, OSBPL7, STK17A, CD3G, ATP8A1, KLF2, CD27, COA1, CXCR5, EPB41, GTF2E1, SLAMF1, TMC6, UBA7, MSN, ALG8, CD79A, UHRF1BP1L, MDFIC, TARBP1, MICB, B3GLCT, CASP2, SELL, EMB, CD37, ABCD4, AP4B1, TTBK2, FAM65B, TMEM5, ANKDD1A, WIPF1, NKRF, SEPT6, IL10RA, SLC25A33, LMO2, DUSP2, NACA, SCIMP, ZSCAN29, RNASET2, RFFL, CD96, CD79B, DGKE, SGSM2, FN3KRP, ZNF700, TBC1D23, PIK3IP1, SP140, DEF6, EVI2A, CARD11, HNRNPA1, RNF43, HLA‐DMB, CD5, TBC1D10C, SEPT1, ZBTB46, S100PBP, EIF4H, NELL2, TMED8, MLLT10, CASP1,TESPA1(171)	Breast cancer	ST	[108]
TLS imprint signature	IGHA1, IGHG1, IGHG2, IGHG3, IGHG4, IGHGP, IGHM, IGKC, IGLC1, IGLC2, IGLC3, JCHAIN, CD52, CD79A, FCRL5, MZB1, SSR4, XBP1, TRBC2, IL7R, CXCL12, LUM, C1QA, C7, APOE, PTLP, PTGDS, PIM2, DERL3(29)	Renal cell carcinoma	10x Visium	[109]

## 4 Prospects

The unique ability of SRT to simultaneously preserve the spatial context of cells and provide information on gene expression makes it highly suitable for characterizing solid tumors. Whether it is interrogating the progression of malignant cells, understanding the distribution of various cell types in tissues, tracing cell lineage and population evolution, exploring the interaction of malignant cells with surrounding stroma and immune cells, or identifying and localizing special spatial structure TLS in the tumor microenvironment, these are highly relevant to tumorigenesis and progression, tumor therapy response, and patient prognosis. For its uniqueness and irreplaceability in real‐time spatial information acquisition, we expect the SRT technology could be further optimized to overcome the limitation of this technology, such as improving spatial resolution without compromising throughput, addressing potential transcript spreading by in situ methods, providing 3D spatially resolved transcriptomic information, and refining database and data analysis methods. These optimizations will allow researchers to integrate macroscopic and microscopic perspectives in exploring precision cancer therapeutics.

With the maturation and diffusion of SRT technology, there is an increasing number of studies that combine SRT with other spatial‐omics analyses for cancer research applications, including spatial genomics,^[^ ^50^, ^111^ ^]^ spatial proteomics,^[^ ^86^, ^112^ ^]^ spatial metabolomics,^[^ ^113^, ^114^ ^]^ and spatial T cell receptor (TCR) sequencing,^[^ ^115^ ^]^ etc. The skyscrapers of spatial multi‐omics are rapidly emerging. For example, high‐resolution multi‐omics characterization of heterogeneity within colorectal tumors was achieved using spatial genomics Slide‐DNA‐seq analysis in combination with Slide‐seqV2 analysis, revealing distinct sets of genes associated with clone‐specific genetic aberrations, local tumor microenvironment, or with both.^[^ ^50^ ^]^ In bladder cancer, spatial proteomics together with SRT was used to identify significant co‐localization between CDH12^+^ epithelial cells and immune cells, especially T cells. Moreover, bladder cancer patients rich in CDH12 had excellent responses to immune checkpoint therapy, providing strong evidence for the design of biomarker guided clinical trials.^[^ ^112^ ^]^ In addition, spatial metabolomics and spatial proteomics combined with SRT analysis elucidate the tumor‐host cell interaction, and well reveal the spatio‐temporal alterations of transcriptional heterogeneity and regional hypoxia metabolism in glioblastoma (GBM). It also provides insights into inter‐patient heterogeneity and emphasizes the necessity and importance of personalized treatment for GBM patients. And the spatial metabolomics technology AFDESI‐MSI, spatial lipidomics technology MALDI‐MSI, and 10x Visium were jointly used to explore the microenvironment of gastric cancer. Based on spatially matched metabolic profiles, lipid profiles, and gene expression profiles, Sun et al. visualized the levels and distribution of metabolites, lipids, and related gene expression in tissues, and confirmed the role of arginine, proline, fatty acids, and phospholipids in gastric cancer progression.^[^ ^113^ ^]^ In addition, the spatial epigenomics technologies Spatial‐CUT&Tag,^[^ ^116^ ^]^ Spatial‐ATAC‐seq,^[^ ^117^ ^]^ and Epigenomic MERFISH,^[^ ^118^ ^]^ and the spatial translatomics technology RIBOmap,^[^ ^119^ ^]^ which are also great assistants to SRT technologies,^[^ ^120^ ^]^ are becoming powerful tools for analyzing real‐time cellular states in tissue environments, pending large‐scale application in cancer research. These additional omics data will break the limitations of the type of molecular data obtained by SRT technology and deepen our understanding of tumorigenesis, tumor development, tumor microenvironment heterogeneity, and personalized anticancer therapies in multiple dimensions. A spatial multi‐omics strategy centered on SRT technology will benefit clinical practice and improve the prognosis for cancer patients.

## Conflict of Interest

The authors declare no conflict of interest.
